# Snapshot Mueller spectropolarimeter imager

**DOI:** 10.1038/s41378-023-00588-y

**Published:** 2023-10-07

**Authors:** Tianxiang Dai, Thaibao Phan, Evan W. Wang, Soonyang Kwon, Jaehyeon Son, Myungjun Lee, Jonathan A. Fan

**Affiliations:** 1 Department of Electrical Engineering, Stanford University, Stanford, CA 94305 USA; 2 Equipment R&D Team 4, Mechatronics Research, Samsung Electronics Co., Ltd, Gyeonggi-do, 18848 Republic of Korea

**Keywords:** Micro-optics, Optical sensors, Nanophotonics and plasmonics

## Abstract

We introduce an imaging system that can simultaneously record complete Mueller polarization responses for a set of wavelength channels in a single image capture. The division-of-focal-plane concept combines a multiplexed illumination scheme based on Fourier optics together with an integrated telescopic light-field imaging system. Polarization-resolved imaging is achieved using broadband nanostructured plasmonic polarizers as functional pinhole apertures. The recording of polarization and wavelength information on the image sensor is highly interpretable. We also develop a calibration approach based on a customized neural network architecture that can produce calibrated measurements in real-time. As a proof-of-concept demonstration, we use our calibrated system to accurately reconstruct a thin film thickness map from a four-inch wafer. We anticipate that our concept will have utility in metrology, machine vision, computational imaging, and optical computing platforms.

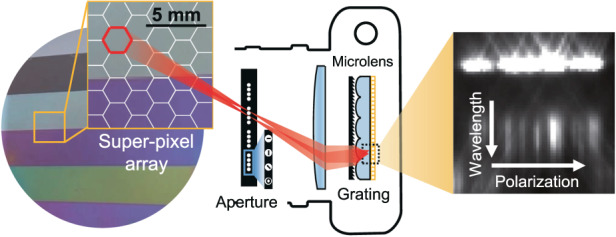

## Introduction

The measurement of the spectral and polarization response of an object given an incident light source is foundational for a broad range of sensing, imaging, and metrology applications. In medicine, spectral- and polarization-based imaging can be used to quantify mechanical stresses and chemical compositions within cells and tissues^1^. In agriculture and the food industry, hyperspectral imaging can inform the health of plants and freshness of food^2^. Infrared polarimetry and spectroscopy are the basis for many remote sensing and machine vision tasks, from identifying the chemical makeup of an object^3,4^ to differentiating between natural and man-made features within a scene^5^. For metrology methods such as ellipsometry, the polarization and spectral analysis of thin film structures is the industry standard for quantifying refractive indices and geometric structure parameters^6–8^.

For many applications, fast acquisition times are required due to the need for high throughput imaging or because the object properties are time-varying. To address this need, a variety of snapshot optical imaging system configurations have been developed that capture the spectral and polarization properties of objects with a single image sensor exposure^9,10^. Snapshot hyperspectral systems have been realized with the incorporation of dispersive elements^11^, computational imaging apertures^12^, and wavelength filters^13^ in the optical system. Snapshot imaging polarimetry has been accomplished by the use of schemes based on division-of-amplitude^14^, division-of-aperture^15^, and division-of-focal-plane^16^ architectures. Snapshot hyperspectral polarimetry systems that combine concepts from hyperspectral and polarimetry imaging^17–19^ have been developed to capture full polarization-spectrum hypercube information.

The most comprehensive linear optical characterization of an object involves hyperspectral Mueller-polarimetry imaging, where the full polarization response of an objective is recorded as a function of illumination polarization and wavelength. Such an imaging capability represents the gold standard in advanced metrology applications and is the basis for instruments such as imaging ellipsometers^20–23^. It remains a challenge to realize these systems with snapshot functionality due to the large number of wavelength and polarization parameters that need to be simultaneously measured. The systems that come closest are non-imaging hyperspectral Mueller polarimeters, which use amplitude modulation in the spectral domain to encode generated polarization states and either spectral^24^ or spatial^25^ modulation to encode polarization and spectral analysis information.

**Fig. 1:**
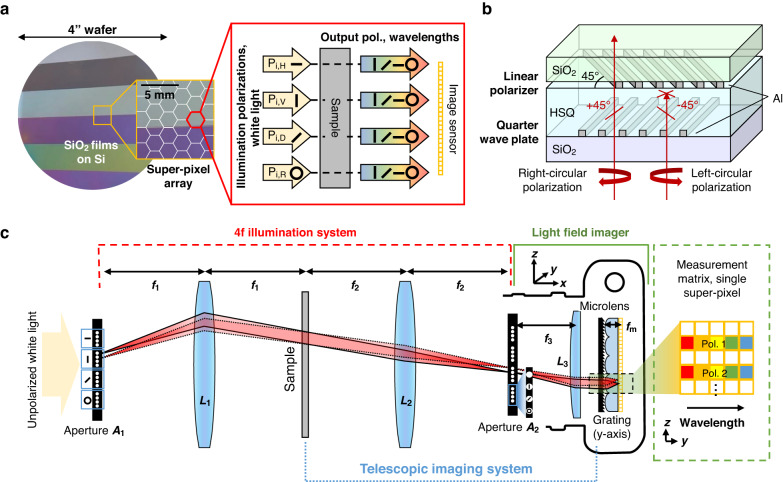
Overview of the snapshot Mueller spectropolarimetry imaging system. **a** Image of a 4” wafer to be analyzed by the system. The imaging system produces a 30 × 40 super-pixel map of the wafer, and within each super-pixel, the full polarization response for 11 wavelength channels (550–800 nm) is recorded. **b** Polarization analysis is achieved with linear- and circular-polarized nanoplasmonic filters. The schematic shows a broadband circular polarization filter comprising a vertical stack of linear polarizer and quarter waveplate devices. **c** Schematic of the imaging system with light paths from two illumination pinhole apertures illuminating a particular spot on the sample. The sample is placed at the Fourier plane of a 4*f* system (Lenses *L*_1_ and *L*_2_) for illumination and is imaged using telescopic and light field imaging systems. Apertures *A*_1_ and *A*_2_, which contain pinhole polarization filters, serve as polarized illumination sources and analyzers, respectively. Imaged rows within each super-pixel correspond to the wavelength response for a particular illumination and analyzed polarization state

We report a snapshot Mueller spectropolarimeter imaging system that can record the Mueller matrix response of an object for visible wavelengths at 1200 spatial positions. It is configured to illuminate and analyze flat objects supporting specular interactions, making it ideally suited for metrology applications with flat substrates. The system as demonstrated images four-inch wafers and uses nanostructured plasmonic polarizers to perform the generation and analysis of polarization states with a horizontal, vertical, diagonal, and right-handed circular polarization (RCP) basis (Fig. 1a). The plasmonic polarizers comprise single- and bi-layer nanoscale aluminum structures that can be tailored to filter linear and circular polarized light with high selectivity and wide bandwidth (Fig. 1b). As a proof-of-concept demonstration, we use our calibrated apparatus to characterize silicon dioxide thin films on a silicon wafer and measure thin film thicknesses to be within 2% of ellipsometry values.

In addition to featuring the unprecedented capability of snapshot Mueller spectropolarimetry imaging, our system features other noteworthy distinctions. First, data readout is highly interpretable, and in a perfectly aligned system, the Mueller matrix for a given wavelength and spatial position can be directly read off of the image sensor with no need for data reconstruction. Even in an imperfectly aligned system, the data remains sufficiently interpretable to enable data denoising and calibration to be performed using only matrix multiplication operations at high speeds. Second, wavelength and polarization information are independently recorded, and unlike methods that encode polarization information using spectral amplitude modulation, no assumptions about the spectral dispersion properties of the sample are required. Third, polarization control and analysis are enabled by nanostructured polarizers fabricated on a chip, enabling multi-aperture arrays capable of supporting customized polarization responses and operating wavelength ranges. Fourth, the light field camera system with analyzing polarization filters can itself serve as a compact hyperspectral polarimeter imaging system.

## Results

### System architecture

A schematic detailing the optical system architecture is shown in Fig. 1c. The schematic is illustrated for a transmissive sample for clarity but readily applies to reflective samples without loss of generality. Conceptually, our imaging Mueller spectropolarimeter comprises a superposition of three optical systems that work synergistically to enable the extreme multiplexing capabilities of our system: an illumination system based on an array of pinhole apertures coupled to a symmetric 4*f* system (*L*_1_ and *L*_2_ are identical), a telescopic imaging system for large area wafer imaging, and a light field camera for capturing multiple images of the wafer with different polarization responses. The sensor pixels under individual microlens, which we term a super-pixel, record the wavelength and polarization response from a specific point of the sample (Fig. 1c, right). A calibration algorithm, based on a customized neural network architecture trained with calibration data, accounts for misalignments and component imperfections. Optical component metrics used in the experimental system are listed in Table S1 and labeled in Fig. S1.

The 4*f* system for illumination maps fields from the front focal plane of the first lens (i.e., the illumination plane) to the back focal plane of the second lens (i.e., the analyzer plane). We consider an array of 16 pinhole apertures at the illumination plane, each of which serves as a distinct illumination source. Placement of broadband polarization filters with linear- or circular-polarized responses at each pinhole leads to a set of 16 polarized illumination sources. At the analyzer plane, a second set of 16 pinhole apertures, each containing unique broadband polarization filters, is placed such that fields from one pinhole at the illumination plane register to one pinhole at the analyzer plane. The light transmitted through an individual analyzer pinhole, which we term a polarization channel, therefore corresponds to a light source paired with a unique combination of illumination and analyzer polarization filters.

The sample is placed at the Fourier plane of the 4*f* system and is imaged using a telescopic system, which enables the imaging of large sample areas. In this scheme, the telescope objective is the second lens of the 4*f* system (*L*_2_) and the eyepiece is *L*_3_ and placed within the light field imager. Demagnification of the sample image at the sensor plane is set to *f*_2_/*f*_3_, where *f*_2_ is the focal distance of *L*_2_ and *f*_3_ is the focal distance of *L*_3_. At the sample position, light from each pinhole at the illumination plane collimates to a plane wave spanning the sample width with a distinctive incidence angle that corresponds to the pinhole position (Fig. S2). In this manner, the illumination sources at the illumination plane interact with the sample in an independent and angle-multiplexed manner.

Finally, light transmitting through the analyzer pinhole apertures is recorded using a modified light field camera, which incorporates a microlens array one microlens focal distance away from the sensor plane^26,27^. The microlenses serve as miniaturized imaging systems that map light field information from the aperture, which in our case is the set of analyzer pinholes, to unique pixels in the sensor array. They also serve to concentrate light spanning the microlens cross-sectional area and enhance the measured signal-to-noise by a factor of *N*, where *N* is the number of sensor pixels under each microlens. A diffraction grating placed just above the microlenses disperses light to the +1 diffraction order to enable hyperspectral imaging for each polarization channel. The light from higher orders carries much less energy, and its effect can be removed using our calibration algorithm. Our spectropolarimetry system therefore is a division-of-focal-plane system that leverages the synergistic coupling of sample illumination in the Fourier plane, in which polarization channels are multiplexed by wavevector angle, with light field imaging, in which waves with different wavevector angles are independently imaged. More details pertaining to the design and operation of the light field imager are in the Supplementary Section.

To optimize the spectral resolution and bandwidth of the system while maintaining minimal crosstalk between super-pixels and polarization channels, we derive basic design guidelines. These guidelines assume that the optical components operate in the scalar diffraction limit and that the diffraction grating transmits light to only the 0 and +1 diffraction orders. First, the microlens diameter (*d*_m_) is specified based on the desired spatial resolution of the system, which is set to *d*_m_*f*_2_/*f*_3_. The microlens focal length (*f*_m_) is then specified to minimize the focused spot size onto the image sensor. Spot size is determined by two factors, the diffraction limit and off-axis aberrations. Using diffraction theory to determine the diffraction-limited spot size and ray simulations to quantify spot sizes for off-axis illumination, optimal combinations of *d*_*m*_ and *f*_*m*_ that minimize spot size can be computed (Fig. S5d).

To maximize spectral bandwidth, the spatial dimensions of exposed image sensor pixels within an individual super-pixel should span *d*_m_, which maximizes the super-pixel area without introducing crosstalk between super-pixels. Given *λ*_max_, the longest wavelength processed in the system, and *n*_m_, the microlens refractive index, the grating pitch (Λ) is fixed using basic relations from diffraction theory and is *λ*_max_*f*_m_*/d*_m_*n*_m_. With set grating and microlens parameters, the spectral resolution (*δλ*) at a given wavelength (*λ*) is *δλ* = Λ*λ/d*_m_. This expression assumes the sensor pixel dimensions match the diffraction-limited microlens focusing spot size.

With the microlens and grating parameters determined, the pinhole analyzer aperture sizes can be specified. For light passing through a finite-sized aperture, *L*_3_ will produce a beam that increases in divergence with increased aperture size. This divergence will lead to an angular distribution of plane waves incident onto the grating and microlens, leading to blurring and reduced spectral imaging resolution. To minimize this effect, we specify the maximum pinhole aperture size such that beam divergence from *L*_3_ produces a blurring length scale at the imaging sensor that is less than the diffraction-limited spot size: *d*_a_ = *λf*_3_*/d*_m_. The spacing between the apertures should be sufficiently large to minimize crosstalk between neighboring polarization channels on the sensor, but sufficiently small so as to not waste pixels on the sensor. We separate neighboring polarization channels by one full row of sensor pixels in a diffraction-limited system by specifying the center-to-center spacing between apertures to be: 2*λf*_3_/*d*_m_.

### Experimental implementation

**Fig. 2:**
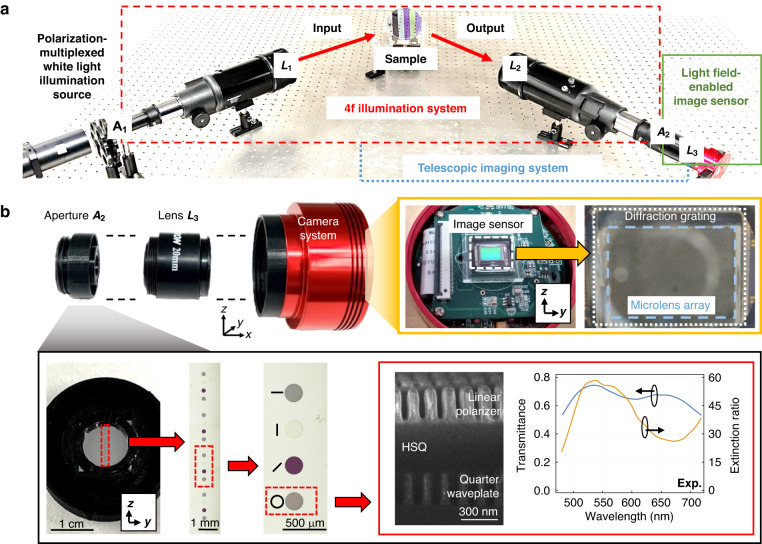
Experimental system. **a** Experimental spectropolarimetry imager setup with delineation of superimposed optical system modules. **b** Experimental images of the light field-enabled image sensor. The camera system comprises polarized pinhole apertures (*A*_2_) and a collimating lens (*L*_3_), and a diffraction grating and microlens array are mounted over the image sensor. The 200 µm-diameter pinhole apertures comprise sets of linearly polarized and RCP filters made of nanostructured, high-aspect-ratio aluminum ridges. A cross-sectional view of a representative RCP filter shows a quarter wave plate and linear polarizer nanoridge devices separated by a spin-on-glass planarization layer. Experimentally measured transmission and extinction of the linear (Supplementary Section) and RCP (shown) filters show high transmittance and extinction over a broadband

The experimentally implemented system is shown in Fig. 2a and records a 40 × 30 super-pixel image of the sample. Each super-pixel corresponds to the integrated optical response of a 3.8 mm diameter area on the sample and includes the full Mueller response for 11 wavelength channels for wavelengths ranging from 550 to 800 nm. A 10-W Tungsten halogen white light source is used for illumination and the exposure time is 17 ms and is limited by the camera frame rate. Our ability to utilize such fast exposure times in spite of our use of pinhole apertures is due to the signal-to-noise enhancement from light concentration mediated by the microlenses. Telescope objective lenses are used to achieve illumination beam widths suitable for wafer-scale imaging. The angle of incidence on the sample is 45 degrees. We note that the system configuration is specified in large part to the availability of specific components and is not fully optimized using the design framework above.

A detailed view of the light field imager, which comprises the analyzer pinhole apertures (*A*_2_), collimating lens (*L*_3_), and functionalized image sensor, is presented in Fig. 2b. Modifications to a commercial image sensor are performed by first removing the protective cover glass over the sensor array followed by mounting of the microlens array and diffraction grating. The pinhole aperture array comprises four sets of four pinholes each for a total of 16 pinholes. Each pinhole is 200 microns in diameter, and each pinhole set contains polarization filters with broadband horizontal, vertical, diagonal, and RCP filtering functionality.

The broadband polarizers consist of nanoplasmonic aluminum structures featuring subwavelength-scale nanoridges. Metallic nanoridge arrays are well known to support the selective filtering of linearly polarized light^28–30^, and they can also be configured to serve as broadband quarter waveplates^31^. The horizontal, vertical, and diagonal polarizers consist of a single-layer linear polarizer structure rotated at different orientations. To enhance transmission and bandwidth, the polarizers are clad in silicon dioxide but contain air gaps between the ridges. The RCP filter comprises a quarter waveplate nanoridge device vertically stacked with a linear polarizer, separated by a dielectric layer (Fig. 2b). We consider plasmonic polarization optics, as opposed to those based on dielectrics^32^, due to ease of fabrication and their ability to support high polarization selectivity over relatively wide bandwidths and incidence angles.

Device fabrication is performed by first depositing an aluminum thin film onto a glass substrate, followed by electron beam lithography patterning and reactive ion etching. As the aperture dimensions are microscale, fabrication of the full set of analyzer filters involves a total patterned area of less than a square millimeter. The multi-layer RCP filter is created by first fabricating the quarter wave plate, planarizing the device with spin-on-glass, and then fabricating the linear polarizer. A cross-sectional image of a representative RCP filter combining the linear polarizer and quarter waveplate, without the top cladding layer, is in the inset of Fig. 2 and shows vertically stacked periodically spaced nanoscale aluminum ridges with vertical side walls. The experimental transmittance and polarization selectivity of the linear (Fig. S8g) and RCP (Fig. 2) filters at normal incident angles indicate high transmittance and selectivity across broadband. Deviations between experimental and theoretical performance are attributed to experimental variations in metal quality, sidewall roughness, and thin film thickness. The filters for the polarization state generator are fabricated in the same manner as those for the imaging aperture. Additional details pertaining to system assembly, filter design, and filter fabrication are provided in the Supplementary Section.

**Fig. 3:**
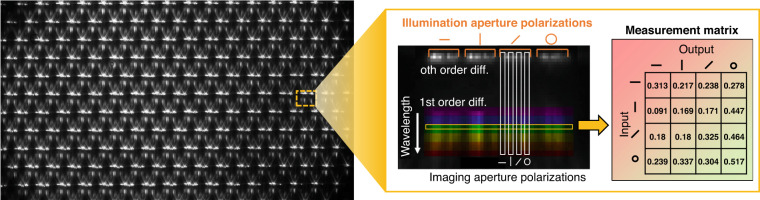
Representative raw image sensor recording showing an array of super-pixels. Each super-pixel contains 16 columns representing different illumination and imaging aperture polarization states, and the rows contain spectral information. The resulting measurement matrices for each wavelength channel can be directly read off as rows on the sensor (yellow box) and converted into Mueller matrices

A representative raw spectropolarimetry image recorded on the sensor shows a two-dimensional array of super-pixels (Fig. 3), with each super-pixel corresponding to light processed from individual microlens. The image as shown is rotated by 90° relative to prior schematic illustrations for clarity. An individual super-pixel comprises sixteen columns that each contain information about a specific incidence and analyzed polarization state. The top row within the super-pixel corresponds to zeroth-order diffracted light while the lower part of the image contains 11 rows from first-order diffracted light spanning 550 to 800 nm. An individual row from the lower part of the image produces our measurement matrix for a given wavelength bin within our experimental polarization basis. These measurements can be readily translated to Mueller matrices using transformations discussed in the Supplementary Section.

### System calibration

**Fig. 4:**
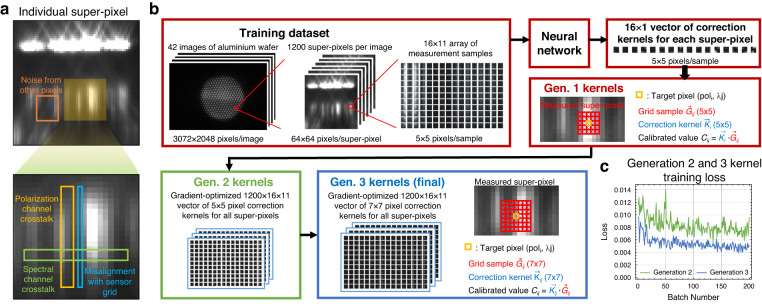
Image sensor calibration procedure. **a** Noise from our experimental system arises from random noise and crosstalk between super-pixels (orange box), polarization channel crosstalk (yellow box), spectral channel crosstalk (green box), and misalignment errors between focal spots and the image sensor grid (blue box). **b** The calculation of denoising kernels **K** is performed in three steps. First, a calibration neural network is trained in which the input is an individual 64 × 64 super-pixel image and the output is a set of 16, 5 × 5 correction kernels, each tailored for a particular polarization channel in the super-pixel. Second, local gradient descent is used to produce unique 5 × 5 correction kernels for all combinations of wavelengths and polarizations for all 1200 super-pixels. Third, local gradient descent is used to produce unique 7 × 7 correction kernels for all pixels. To calibrate a specific measured pixel on the image sensor, a 7 × 7-pixel image centered at the pixel of interest is multiplied by the corresponding correction kernel (blue box, inset). Network training is based on training data obtained from measurements with an aluminum wafer reference. **c** Visualization of the loss curves during gradient-based optimization of second and third generation kernels

In an ideally implemented system, the measurement matrix can be directly read off and used to accurately compute Mueller matrices. However, in an experimental system, misalignments between system components and physical imperfections within the components themselves lead to noise in the super-pixel images. The wave nature of light also prevents the ideal readoff of independent wavelength and polarization information from an individual sensor pixel, as the center of the Airy disk from a focused beam contains 84% of the total power and the remainder of the power resides in concentric rings around the disk. Collectively, these factors produce systematic nonidealities that manifest as reduced signal-to-noise and crosstalk within and between polarization and wavelength channels (Fig. 4a). Additionally, misalignment between the microlens focal spots and the discrete sensor grid (i.e., sub-pixel misalignment) can lead to detection sampling errors. Due to the complexity of this error and its combination of systematic and random sources, conventional approaches to denoising, such as the fitting of a global point spread function, are not effective (Fig. S14).

To correct these noise sources, we consider an approach where a given sensor pixel within a super-pixel, which targets a specific wavelength band and polarization measurement state in an ideally aligned system, is corrected using a tailored denoising kernel, **K**. Given **P**, a pixel sensor image centered at the pixel of interest with the same dimensionality as **K**, the calibrated measurement value is *M* = **K** *·* **P**. This approach assumes that nonidealities in our experimental system produce local variations in the point spread function on the image sensor. It also builds on our observation that the optical system is linear and the true intensity values at a given pixel must be a linear combination of intensity values from surrounding pixels within the point spread function.

In a naive denoising approach, denoising kernels can be directly learned for every wavelength band and polarization measurement state within a super-pixel (11 × 16 = 176 kernels) for every super-pixel (30 × 40 super-pixels), using calibration data taken from measurements from a known reference wafer. Such a direct learning approach with limited training data is not effective and will lead to significant overfitting. We instead take a multi-step approach in which we first consolidate data from all super-pixels and use a deep network to learn first generation 5 × 5 kernels with limited accuracy, discussed in more detail below, which overcomes the overfitting issue and enables the learning of correlated noise between super-pixels (Fig 4b). Second-generation kernels, also with dimensions of 5 × 5, are tailored to of each wavelength band and polarization state of each super-pixel for a total of 11 × 16 × 1200 kernels. These kernels are computed by performing local gradient-based optimization on first generation kernels using the original calibration data, and intensity information from the first generation kernel learning process is incorporated to correct illumination heterogeneity in the system. The third generation kernels, which are the final kernels used for calibration, are computed by padding the second generation kernels to 7 × 7 dimensions and performing local gradient-based optimization with the original calibration data.

To produce first generation kernels, we utilize a customized neural network architecture in which the input is an individual 64 × 64 super-pixel image and the output is a set of 16 denoising kernels that are tailored for each polarization channel. This concept builds on the observation that many sources of noise, such as component misalignment, produce systematic errors in the recorded image that lead to shared noise characteristics between different super-pixels. The neural network architecture (Fig. S11a, b, d) bears unique properties that differ from typical convolutional neural networks used in classical computer vision tasks^33–35^. First, the network is specified to be relatively shallow to minimize overfitting and provide better stability with the use of a limited training dataset. Second, the network explicitly accounts for sub-pixel misalignments by upscaling the input image by 8× with bilinear sampling. Third, the network explicitly incorporates translation symmetry such that translational shifts of whole- and sub-pixel shifts within the input image lead to corresponding shifts in the outputted kernels. For the training process, a total of 42 images were taken of an aluminum reflection mirror with different combinations of polarizers and spectral filters placed at the light source and analyzers. 232 randomly selected super-pixels from each image were selected to formulate the training set, yielding a total of 42 × 232 = 9744 super-pixels for the training set. A representative training loss curve of first generation kernels is shown in Fig. S11e and indicates good convergence over the course of network training. Loss curves for second and third generation kernels averaged over all super-pixels are shown in Fig. 4c and also indicates good convergence.

### Calibrated measurement

**Fig. 5:**
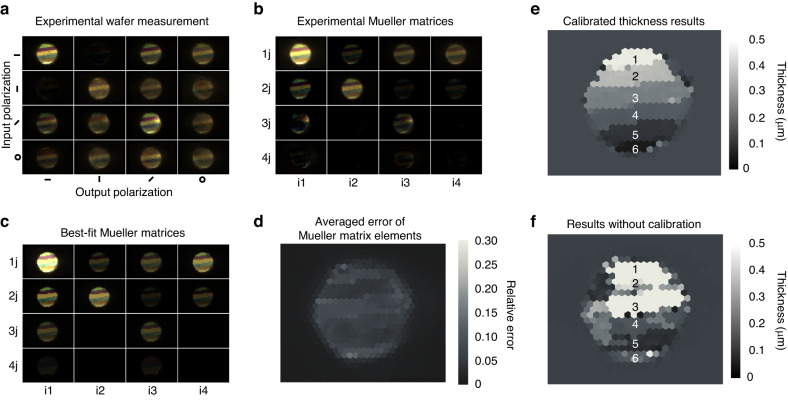
Benchmark imaging results. **a** Experimental images of measurement matrix data from the 4” wafer in Fig. 1a comprising silicon dioxide thin films on a silicon wafer. **b** Corresponding computed Mueller matrix data. **c** Mueller matrix data based on a best-fit thin film model. **d** Averaged relative error of Mueller matrix element of each super-pixel. **e** Calibrated, experimentally measured silicon dioxide thickness map based on the best-fit thin film model. **f** Silicon dioxide thickness map based on the application of the best-fit thin film model to uncalibrated experimental data

To demonstrate the capabilities of our system, we perform Mueller spectropolarimetry imaging on the 4” wafer shown in Fig. 1a. Image calibration involves dot product multiplication of third-generation kernels with the super-pixel images and takes 200 ms using standard CPU hardware (Intel core i7-8700K). As a point of comparison, the direct use of a neural network to perform super-pixel calibration over a wafer scale would take 15 min with the same hardware. Faster and parallelized matrix computations can be achieved using graphics processing unit (GPU) hardware. The calibrated measurement matrices at all points on the wafer are visualized in Fig. 5a, where the spectral response at a given super-pixel is encoded by a standard color vision response. The smooth and consistent color within each silicon dioxide band on the wafer, together with polarization features such as the lack of signal for cross-polarized input and analyzed polarization states for horizontal and vertical polarization, indicate the efficacy of our calibration method.

The measurement matrices can be used to compute the corresponding Mueller matrices, shown in Fig. 5b, using methods discussed in the Supplementary Section. The absolute error maps for each Mueller element in each wavelength band and super-pixel, compared to Mueller elements computed from film values and indices measured at individual regions on the wafer using ellipsometry (Table 1), are presented in Fig. S15. A map of the absolute error from the Mueller matrix elements in each super-pixel, averaged over all matrix elements for all wavelengths, is shown in Fig. 5d. These results display consistently low error with an overall average of 8%. The presence of speckle patterns suggests random super-pixel-specific manufacturing defects in the lens array or grating that cannot be corrected with our calibration method, and their elimination reduces the total average error to 6%.

**Table 1:** Calibrated, experimentally measured silicon dioxide thickness based on the best-fit thin film model, in comparison with values obtained by ellipsometry

	Our results (µm)	Ellipsometer (µm)
1	0.498 ± 0.006	0.497 ± 0.006
2	0.391 ± 0.005	0.392 ± 0.005
3	0.278 ± 0.006	0.288 ± 0.003
4	0.188 ± 0.005	0.187 ± 0.005
5	0.081 ± 0.008	0.086 ± 0.004
6	0.002 ± 0.002	0.004 ± 0.001

Numbers correspond to labeled regions in Fig. 5d

These experimental data are used to calculate the thickness of silica at each super-pixel by minimizing the absolute error between the computed experimental Mueller matrices and those corresponding to a thin film fitting model. The resulting best-fit thin film model, visualized as Mueller matrix elements, is shown in Fig. 5c and agrees well with the experimental results. The resulting thin film thickness map for the wafer is presented in Fig. 5e and shows clear uniformity within each band of silicon dioxide. Deviations of the thicknesses are computed with respect to pixels that belong to the region in the thickness map. The averaged thicknesses within each silicon dioxide layer are, on average, within 5 nm of ground truth values (Table 1). We note that deviations within the thickness map arise at boundaries between silicon dioxide layers and along the edges of the wafer, due to the limited spatial resolution of our system. The uncalibrated thickness map (Fig. 5f) is inaccurate and indicates the need for our calibration method.

## Discussion

In summary, we present an optical imaging system architecture that is capable of snapshot Mueller spectropolarimetry imaging for flat objects supporting specular interactions. Light from multiple illumination sources is simultaneously imaged by coupling a Fourier optic illumination scheme with a telescopic light field image sensor. Arrays of microscale nanoplasmonic polarization filters at the illumination and analyzer planes enable field-multiplexed imaging of unique polarization responses from the object. The design rules for the system are simple and straightforward, and the combination of a customized calibration neural network architecture together with an interpretable data readout scheme ensures accurate data capture. A proof-of-concept demonstration with a 4” calibration wafer indicates the potential of the system to accurately perform metrology tasks.

There exist multiple immediate opportunities for improving system performance. As discussed, the specifications used in our study are far from optimal, and further component customization can enable the maximization of spectral and spatial system bandwidth. The use of additive manufacturing to directly print a customized microlens array onto the image sensor facet can lead to ideal microlens alignment with the sensor, fully tailorable microlens parameters, and integration of the diffraction grating directly onto the microlens surface. Improvements in experimental filter efficiency can be achieved using optimized fabrication processes, device schemes that feature relatively low metal fill fraction, and device schemes that utilize lower loss metal materials such as silver^31^. Filter device fabrication can be scaled up using a variety of patterning techniques that exceed the throughput of electron beam lithography, including nanoimprint, laser interference, and deep UV lithography. As a metrology tool, incorporating a goniometer stage with our system can enable measurements at different light incidence angles, producing more data for more accurate analysis. Longer term, we envision that our concept can extend to the analysis of microscopic domains through the use of microscope objectives in the 4*f* system and that it can be implemented in other imaging and optical data processing modalities through the utilization of metasurface apertures with more customized optical responses^36–41^. With proper co-design of aperture responses with software, our imaging system can be tailored for tasks as diverse as optical computing and data compression, and it can combine with concepts in computational imaging to enable enhanced imaging capabilities^42^.

## Materials and methods

Methods pertaining to optical system construction, nanopolarizer fabrication, neural network architecture and training for calibration, and additional experimental results are provided in a separate Supplementary Section file.

### Supplementary information

Supplementary Information for Snapshot Mueller spectropolarimeter imager
