# Supplementary material for: Snapshot Mueller spectropolarimeter imager
Source: Microsyst Nanoeng. 2023 Oct 7;9:125. doi: 10.1038/s41378-023-00588-y (PMC10560212; doi:10.1038/s41378-023-00588-y)
Supplement: Supplementary file 1 — Supplementary Information for Snapshot Mueller spectropolarimeter imager [file 41378_2023_588_MOESM1_ESM.pdf]

Supplementary Information for Snapshot Mueller spectropolarimeter imager

Tianxiang Dai[1], Thaibao Phan[1], Evan W. Wang[1], Soonyang Kwon[2], Jaehyeon Son[2], Myungjun Lee[2] and Jonathan A. Fan[1]

[1]Department of Electrical Engineering, Stanford University, 348 Via Pueblo, Stanford, 94305, CA, USA.
[2]Equipment R&D Team 4, Mechatronics Research, Samsung Electronics Co., Ltd., Gyeonggi-do, 18848, Republic of Korea.

Contributing authors: txdai@stanford.edu; mvarlaro@stanford.edu ; wangevan@alumni.stanford.edu; sy13.kwon@samsung.com; jw94.sung@samsung.com; myung01.lee@samsung.com; jonfan@stanford.edu;

Contents

S1 System metrics **3**

S2 Optical system architecture **3**
- S2.1 4f illumination system . . . 4
- S2.2 Light field camera . . . 5
- S2.3 Microlens design and simulation . . . 7
- S2.4 Nanoridge polarizer design . . . 8
- S2.5 Signal-to-noise . . . 12

S3 Systems assembly and device fabrication **12**
- S3.1 Polarization filter nanofabrication and characterization . . . 12
- S3.2 Optical system assembly . . . 13

S4 Measurement matrix to Mueller matrix conversion **14**

S5 Calibration algorithm **15**
- S5.1 Neural network architecture . . . 15

S5.2 Neural network training and evaluation 16

S6 Thin film measurement **18**

S1 System metrics

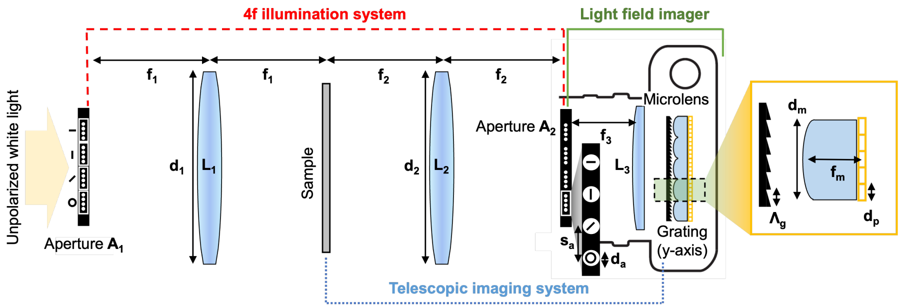

Fig. S1: Diagram of full optical system with labeled design parameters.

The following tables summarize parameters pertaining to the optical system setup and design shown in Figure S1.

Parameter	Value	Description
f_1	400mm	Lens L_1 focal length
d_1	80mm	Lens L_1 diameter
f_2	400mm	Lens L_2 focal length
d_2	80mm	Lens L_2 diameter
d_a	200μm	Polarizer aperture diameter
s_a	400μm	Polarizer aperture pitch
f_3	20mm	Lens L_3 focal length
d_m	190μm	Individual microlens diameter
f_m	250μm	Microlens focal length
d_p	2.4μm	Sensor pixel width
Λ_g	1.67μm	Diffraction grating period

Table S1: System component parameters.

Parameter	Equation	Value	Description
$f_1/\#$	d_a/f_1	$f/100$	System focal ratio
$f_m/\#$	d_m/f_m	$f/1.3$	Microlens focal ratio
d_d	$\lambda f_m/d_m$	0.72μm	Microlens diffraction-limited focus diameter

Table S2: Derived system parameters.

S2 Optical system architecture

In this section, we present a more detailed disucssion of the operating principles behind our optical system.

Parameter	Value	Description
$d\lambda$	35 nm	Wavelength resolution
$\lambda_{\max}$	0.9μm	Maximum wavelength
$f_e/\#$	$f/14$	Effective focal ratio
d_p	120 mm	Parallax-free imaging range

Table S3: Performance parameters.

S2.1 4f illumination system

Imaging Mueller polarimetry requires sixteen distinct images to be recorded at each object position, each corresponding to distinct combinations of illumination and analyzer polarization states. With a 4f illumination system, a point source at the front focal plane of the first lens (i.e., the illumination plane) gets collimated by the first lens, illuminates and interacts with the sample placed between the lenses, and then refocuses to a point at the back focal plane of the second lens (i.e., the analyzer plane). In this manner, the sample placed at the Fourier plane is illuminated by a plane wave spanning the sample diameter. This concept can generalize to an array of sixteen point sources at the illumination plane that produce multiple incident beams onto the object, refocus to an array of sixteen points at the analyzer plane, and are independently imaged by our multi-pinhole imaging system. Polarimetry imaging is readily achieved by placing unique combinations of polarization filters at the illumination and analyzer apertures. The full beam paths from two illumination apertures are shown in Figure S2. Experimentally, L_1 and L_2 are Celestron achromatic refractor telescopes with a focal length of 400 mm and diameter of 80 mm. We note that obscuration of the lens due to mounting reduces the effective aperture to a diameter of about 65 mm.

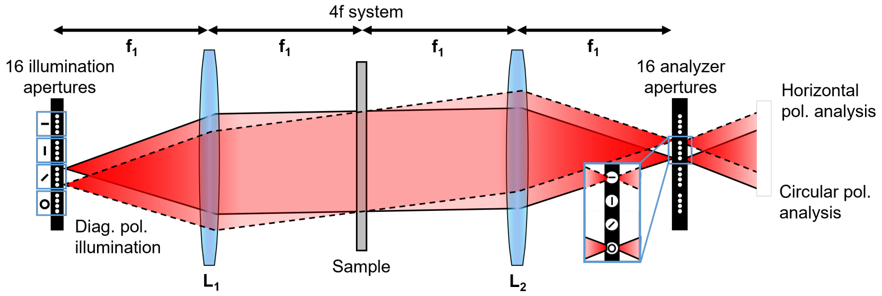

Fig. S2: Diagram of full sample illumination with beam paths from two illumination apertures drawn. These beams correspond to diagonal polarization illumination with horizontal polarization analysis (dashed lines) and diagonal polarization illumination with right circular polarization analysis (solid lines).

With this Fourier-based illumination scheme, light from different pinhole apertures in the illumination plane become plane waves that are incident onto

the sample with different angles. The full angular spread from all illumination pinhole sources is determined by the distance between the two most spatially separated apertures (7.8 mm) and f_1 (400 mm) and is approximately 0.02 radians (1.2°). The angular variation between plane waves incident onto the sample from two adjacent illumination apertures is 0.06°. These narrow angular ranges place tight constraints on the angular alignment of the system and the maximum curvature or wedge angle of the sample to be tested. Our optical configuration is thus best suited for measuring samples that are known to be flat, such as wafers.

We note that in practice, the pinhole apertures in the illumination plane are consolidated to four relatively large area apertures (Figure S3), though they functionally behave as pinholes due to the spatial filtering properties of the 4f system and pinhole analyzer apertures. Our use of relatively large apertures for illumination enables more robust system alignment. The blue box and arrow in the figure represent the mapping of fields from the dashed circular region at the illumination plane with a diagonally-polarized analyzer aperture in the analyzer plane, mediated by the 4f system.

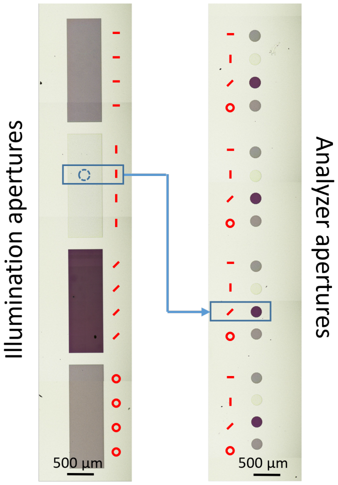

Fig. S3: Images of the polarization filter-functionalized apertures at the illumination and analyzer planes, together with red symbols representing polarization filtering function. The 4f system inverts and maps fields from the illumination aperture to the analyzer aperture.

S2.2 Light field camera

In this subsection, we further elucidate the operating principles of the hyperspectral and polarization-enabled imaging sensor. We note that this sensor system can be used independently as an imaging spectropolarimetry pinhole

camera in which the analyzing apertures serve as imaging pinhole apertures. We first consider an imaging system with a single analyzer aperture. In conjunction with our implementation of a telescopic imaging system, L_3 is placed one focal distance away from the aperture plane and collimates light from the aperture (Figure S4a, top left). In the case where the pinhole aperture is axially aligned with the lens, the collimated light propagates with normal incidence onto the sensor array. By collimating light in this way, image demagnification onto the sensor is fixed to f_2/f_3. For our experimental system configuration, a 20:1 reduction system from sample to image sensor is achieved.

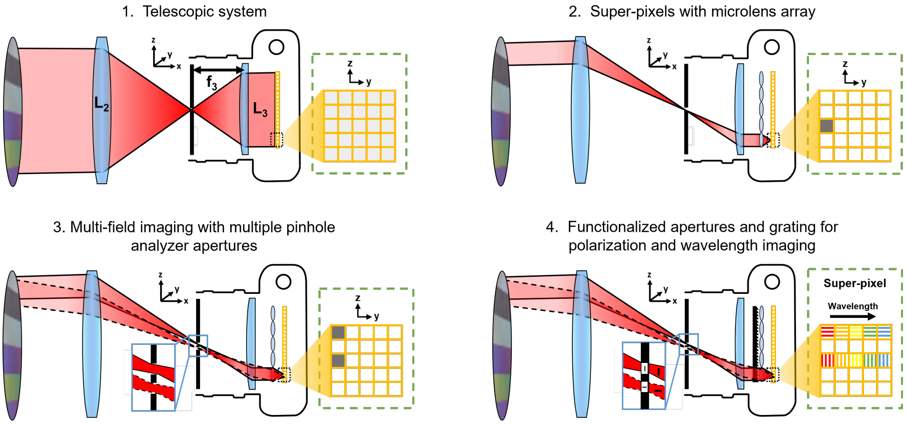

Fig. S4: Conceptual framework for the light field system architecture. 1) The lens (L_3) sets the magnification of the imaging system and collimates light from the pinhole aperture. 2) Addition of a microlens array at the image sensor improves signal-to-noise by performing light concentration. 3) Multiple pinhole apertures coupled to the microlens array enable the simultaneous imaging of multiple fields. 4) The incorporation of polarization filters at the pinhole aperture and a diffraction grating above the microlenses enable polarization- and wavelength-resolved imaging.

We next place a microlens array one microlens focal length away from the image sensor (Figure S4, top right). The incorporation of the microlens array presents a tradeoff between imaging resolution and signal-to-noise: as the light incident onto the microlenses is collimated, the microlenses will focus and concentrate the light to a single pixel, pending that the diffraction-limited microlens spot size is smaller than an individual sensor pixel. In the case where there are N sensor pixels under each microlens ($N = 4900$ for our system), light throughput to the illuminated pixel is enhanced by a factor of N, thereby enhancing the measured signal-to-noise by a factor of N. As the microlenses concentrate light to a single pixel, the resolution of the imaging system is set by the size of the microlenses and is reduced by a factor of N. As a point of comparison, for a system without microlenses and assuming that the measured

signals from a grouping of N pixels are spatially uniform, signal averaging over all pixels will improve signal-to-noise by only a factor of $\sqrt{N}$. The added signal-to-noise enhancement with microlenses is particularly essential in our scheme, as light throughput is limited by our use of pinhole apertures. With the usage of microlenses, the signal-to-noise ratio is sufficiently high even for millisecond exposure times.

The combination of microlenses and L_3 enables light from different pinhole apertures to be imaged onto distinct sets of pixels on the sensor. The concept is illustrated in the bottom left panel in Figure S4 for two pinhole apertures, one axially aligned with the collimating lens and one in a slightly offset position. As shown before, light from the axially aligned aperture collimates into a normally-incident beam, leading to the focusing of light to sensor pixels that are axially aligned with the microlenses. Light from the slightly offset aperture instead collimates to a slightly off-normal direction onto the microlens array, which focuses the light to sensor pixels with slightly off-normal positions at all microlenses. In this manner, the light field camera captures two distinct and independent images.

Finally, polarization analysis is enabled by specifying polarization filters at the apertures (bottom right, Figure S4). In the schematic, broadband vertical and horizontal polarizers are depicted, leading to vertical and horizontal polarization-analyzed images to be recorded onto the image sensor. Sets of devices with distinct polarization responses are nanofabricated in parallel on a single chip, and the total fabricated area is small and dictated by the pinhole aperture dimensions. To obtain hyperspectral information for each polarized image, a diffraction grating oriented along the y-axis (i.e., oriented ninety degrees relative to the pinhole aperture array) is placed just above the microlens plane such that collimated light from each aperture disperses to the +1 diffraction order. The resulting super-pixels on the image sensor comprises rows of pixels, each containing wavelength information for a particular illumination and analyzer polarization state.

S2.3 Microlens design and simulation

To identify suitable microlens designs that support diffraction-limited performance, we perform a systematic array of on-axis and off-axis ray tracing simulations for a wide range of lens parameters and a fixed wavelength of 550 nm. These simulations were performed with the assumption that the microlenses have a spherical surface. Figure S5a shows a representative example of a ray tracing analysis for a microlens with a width of 190 μm and an effective focal length of 250 μm. The short focal ratio causes focusing aberrations: as shown in Figure S5b and S5c, the geometric spot size exceeds the red circle delineating the diffraction limit.

Figure S5d shows the focusing spot size as a function of microlens f /# for various microlens sizes. The points of intersection between the line delineating the diffraction limit and the lines delineating RMS spot size from ray tracing

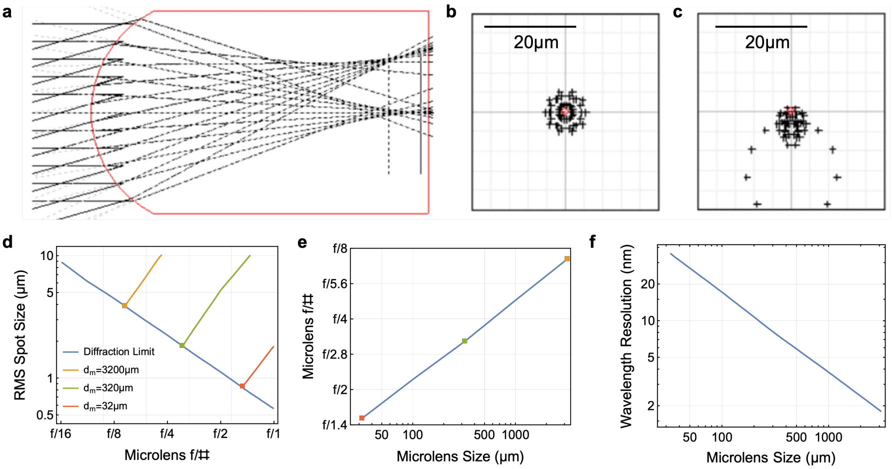

Fig. S5: Microlenses optical characterization. a) Representative on- and off-axis ray tracing simulations of a microlens. b,c) Spot diagrams at the microlens focal plane for (b) normal incidence and (c) 16° incidence. d) RMS spot size as a function of microlens f/# for microlenses with different diameters. The microlens f/#'s corresponding to minimum RMS spot size for a given lens diameter are delineated by circles. e) Optimized microlens f/# versus microlens size based on the circles from (d). f) Wavelength resolution as a function of microlens diameter given a system with a minimized RMS spot size.

represent the ideal condition in which the diffraction-limited and aberration-limited spot sizes are equal. These points of intersection are plotted in terms of relationships between microlens f /# versus microlens diameter (Figure S5e) and wavelength resolution versus microlens size (Figure S5f) to cast these results in terms of ideal design rules for the microlenses.

S2.4 Nanoridge polarizer design

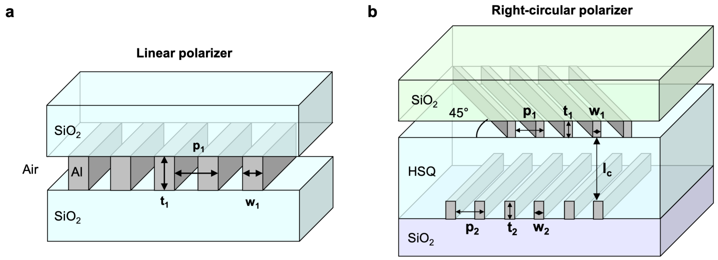

Fig. S6: Diagrams of the (a) linear polarizer and (b) RCP filter with labeled geometrical design parameters.

Figure S6 schematically shows the layout and geometric parameters pertaining to the linear polarization filter and RCP filter. The specific geometric parameter values used in our study are summarized in Table S4. Our notation convention for incidence angle onto the filters is visualized in Figure S7.

Parameter	Value	Description
w_1	75 nm	Linear polarizer ridge width
p_1	150 nm	Linear polarizer ridge period
t_1	240 nm	Linear polarizer ridge thickness
w_2	45 nm	Quarter-wave plate ridge width
p_2	325 nm	Quarter-wave plate ridge period
t_2	240 nm	Quarter-wave plate ridge thickness
l_c	625 nm	Separation between quarter-wave plate and linear polarizer in circular polarizer

Table S4: Geometric parameters for the linear and circular polarizers.

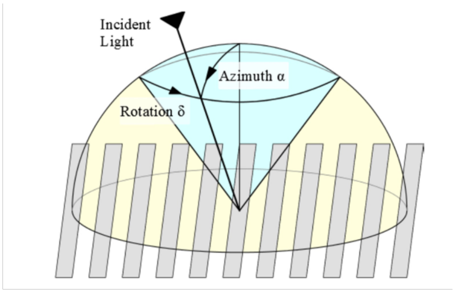

Fig. S7: Parameterization of incidence angle for our nanoridge polarizers.

To design the linear polarizers, we use Reticolo rigorous coupled-wave analysis (RCWA) to simulate devices [1] and perform a parameterization sweep over a wide range of device geometries and incidence angles. We specifically consider a device that is made of aluminum nanoridges, contains air between the lines, and is clad in silicon dioxide at the top and bottom device interfaces. Compared to devices that are fully encapsulated in silicon dioxide or have no top cladding layer, we find that this configuration supports superior transmission due to impedance matching between the silicon dioxide and metal-air layers. We first consider a parameter sweep over nanoridge period and thickness, with g equal to w, for a normally incident 550 nm plane wave. The corresponding maps of transmittance and extinction ratio are shown in Figures S8b and S8c. Based on these maps and fabricability considerations, we choose as our linear polarizer geometry a device featuring a width of 75 nm and gap of 75 nm, which corresponds to a period of 150 nm (marked as stars in the figures).

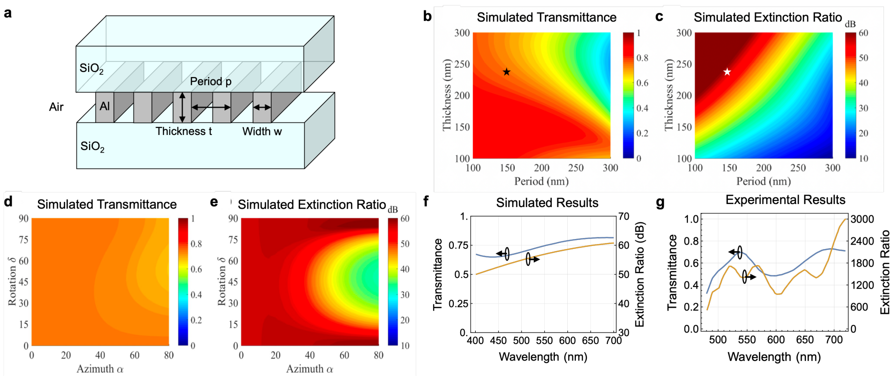

Fig. S8: Linear polarizer design and optimization. a) Schematic of the polarizer layout and material composition. b,c) Simulated (b) transmittance and (c) extinction ratio of periodic aluminum nanoridges with different periods and thicknesses at $\lambda = 550$ nm. d,e) Simulated (d) transmittance and (e) extinction ratio of a linear polarizer (star in (b,c)) at $\lambda = 550$ nm for varying incidence angles. The device has width = 75 nm, period = 150 nm, and thickness = 240 nm. f) Simulated transmittance and extinction ratio of the starred device in (b,c) for normal incidence as a function of wavelength. g) Experimental measurement results for transmission and extinction ratio of the linear polarizer at normal incidence.

We next simulate the wavelength and angular bandwidth of these polarizers to ensure their operation within our system requirements. RCWA simulations of the transmission and selectivity of our polarizer for different incidence angles at $\lambda = 550$ nm are summarized in Figures S8d and S8e, respectively, and indicate high transmission and selectivity for a very wide range of α and δ angles. Figure S8f shows the the transmittance and extinction ratio of this design for a normally incident beam over a wavelength range of 400 to 700 nm and shows an extinction ratio above 50 dB and transmittance above 0.6 for the entire visible spectrum. These broadband and broad angle characteristics are due in part to the non-resonant nature of our device.

Experimentally measured transmittance and selectivity of the linear polarizer is shown in Figure S8g for normally incident visible light. The measurement is performed by collimating a supercontinuum laser source coupled to a monochromator onto the sample and detecting the transmitted light using a standard broadband linear polarizer analyzer and silicon detector. These measurements indicate that the experimental linear polarizer operates with broad bandwidth and high selectivity.

To design the quarter-wave plate (QWP), we use an approach similar to our design of linear polarizers and use RCWA simulations to perform a parametric analysis of aluminum nanoridge structures with varying thicknesses, metal ridge widths, and periods. The results of a representative set of simulations for

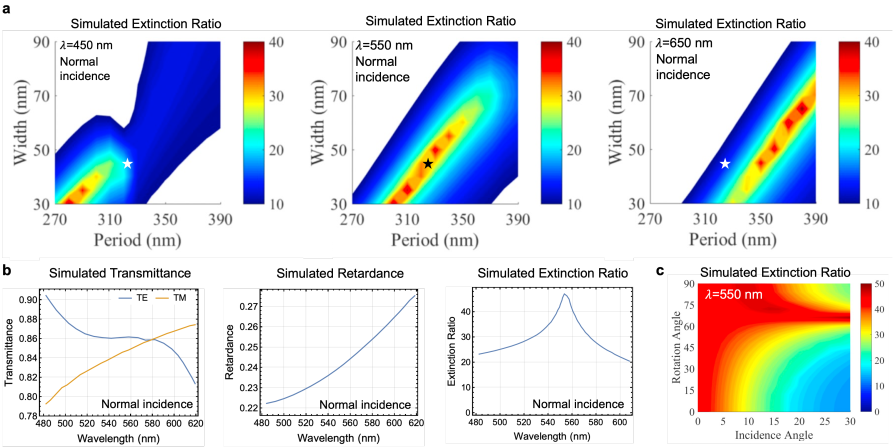

Fig. S9: Quarter-wave plate (QWP) design and simulation. a) Extinction ratio for QWP-based RCP filters with QWP different nanoridge widths and periods (thickness is fixed at 240 nm) at $\lambda = 450{,}550{,}650$ nm. The filter assumes an incident RCP wave and polarization analysis with an ideal linear polarization filter with alignment shown in Figure S6b. b) Simulated transmittance, retardance, and extinction ratio of quarter-wave plate with period = 325 nm, width = 45 nm and thickness = 240 nm as a function of wavelength (star in (a)). The extinction plot assumes implementation as an RCP filter as described in (a). c) Simulated extinction ratio of an RCP filter as a function of incidence angle using the starred QWP in (a).

devices with a thickness = 240 nm, normal incidence, and an operating wavelength of 550 nm is shown in Figure S9a. The extinction ratio as shown is that for a QWP implemented within an RCP filter (Figure S6b) and is computed considering an RCP wave incident onto the QWP followed by polarization filtering with an ideal linear polarizer. Based on our parametric sweeps, we select devices with a period = 325 nm and width = 45 nm for the quarter-wave plate design (marked as stars in the figures). Simulated tranmission and retardance of the QWP and its extinction within an ideal RCP filter over a range of wavelengths is shown in Figure S9b and indicates the non-resonant device supports broadband functionality. Simulation results of the QWP implemented within an ideal RCP filter for different incidence angles are shown in Figure S9c and indicate that the extinction ratio remains high up to azimuth angles of 15°. Reductions in extinction ratios at higher angles are due to diffraction.

Finally, we simulate the combined physical QWP and linear polarizer stack to evaluate the performance of our RCP polarization filter (Figure S10). The performance of these structures are sensitive to the precise spacing between the individual devices because of parasitic electromagnetic coupling between the structures. Figure S10b shows the simulated extinction ratio of the RCP filter as a function of the separation between the QWP and linear polarizer.

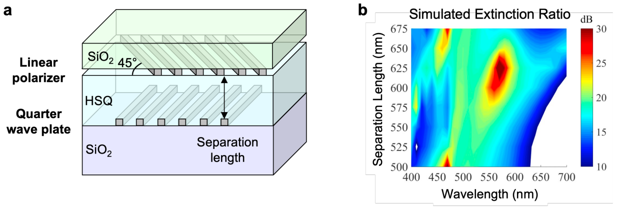

Fig. S10: Circular polarizer filter design. a) Schematic of the RCP filter showing separation length. b) Simulated extinction ratio of the RCP filter as a function of separation between the QWP and linear polarizer.

We find that for a cavity length of 625 nm, the overall extinction ratio of the RCP filter is relatively high across the broad band of simulated wavelengths.

S2.5 Signal-to-noise

To quantify the signal-to-noise of our system, we measure the detected intensity levels at super-pixels with the halogen source turned on and off, averaged over one hundred randomly selected measurement pixels. In arbitrary units, the average detected intensity of pixels with the light source on is 0.15 and and the average detected intensity of pixels with the light source off is 0.00051, giving a signal-to-noise ratio of 300. This indicates that in spite of our use of pinhole apertures, light intensification from the microlenses still enables high signel-to-noise measurements to be made at millisecond speeds and modest light source powers. It additionally indicates that the measured 'noise' in the system is not due to low light levels but is due to a lack of proper calibration.

S3 Systems assembly and device fabrication

S3.1 Polarization filter nanofabrication and characterization

The polarization filter arrays comprise linear and RCP filters and are fabricated in parallel using standard nanofabrication methods at the Stanford Nanofabrication Facility (SNF) and the Stanford Nano Shared Facilities (SNSF). Fabrication is performed on four inch fused silicon dioxide wafers for the devices and on four inch oxidized silicon wafers for cross sectional imaging.

First, the quarter wave plate devices are fabricated. A 240 nm thick aluminum film is deposited on the wafers using electron beam evaporation, which is to be patterned into the quarter wave plate for the RCP filters. A 55 nm thick silicon dioxide layer is then deposited using plasma-enhanced chemical vapor deposition (PECVD) at 350 °C, which serves as a hard mask for thin film patterning. Microscopic alignment markers, used to align the top and bottom devices in the multi-layer RCP filter, are defined on the wafers

using photolithography followed by hard mask etching in an Oxford Capacitively Coupled Plasma (CCP) Etcher and aluminum etching in a Plasma Therm Versaline LL ICP Metal (PT-MTL) Etcher. The quarter waveplate devices are patterned using electron beam lithography (100 keV JEOL 6300 system and positive CSAR-62 ebeam resist) with patterns aligned with the alignment markers, followed by etch steps that follow the CCP and PT-MTL etching procedure from before. The quarter wave plate devices are planarized by spin coating two layers of hydrogen silsesquioxane (HSQ) spin-on-glass on the devices (Corning, FOX-16) at 2000 rpm, followed by baking at 260 degrees Celsius.

Next, the linear polarizers are fabricated. A second aluminum thin film and silicon dioxide hard mask layer is deposited as before. The linear polarizers are fabricated using aligned electron beam lithography and etching as before. To encapsulate the linear polarizers with silicon dioxide while maintaining air spacers between the nanoridges, PECVD silicon dioxide deposition is used and a 300-nm-thick layer is grown. The wafer is diced into chips using the DISCO wafer saw.

To characterize the linear filters, RCP filters, and quarter wave plates, the devices are mounted on a set of rotation and translation stages. A supercontinuum source (NKT Photonics) coupled to a monochrometer is used as the light source and is collimated onto the sample at a desired incidence angle. A polarization generator and analyzer comprising commercial linear and quarter waveplates is used to specify the incident and analyzed polarization states. Light is detected using a silicon photodetector.

S3.2 Optical system assembly

To fabricate the light field imaging system, we modify a commercial ASI178MM monochrome CMOS camera (company is ZWO ASI). First, the cover glass is removed by using a 50 W Epilog laser cutter to decompose the epoxy bonding the cover glass to the image sensor package, followed by careful cover glass removal with a razor blade. Second, the microlens array (385-μm-thick Microlux Fly's-Eye lens sheets with 133 lenses per inch) is created by dicing pieces into precise dimensions using a DISCO wafersaw, followed by manual deburring with a razor blade. Third, the microlens array is bonded to the image sensor using a Karl Suss contact mask aligner for alignment and Norland Optical Adhesive 75 as the adhesive. Fourth, 8 mm x 6 mm diffraction gratings (Dynasil) are bonded to the microlens array by use of a custom jig, optical adhesive, and the Karl Suss contact mask aligner. Bonding is performed at overlapping grating and microlens array regions that are away from the image sensor. To assemble and align the polarization filters with the halogen source and light field image sensor, custom holders were made using additive manufacturing.

S4 Measurement matrix to Mueller matrix conversion

The Mueller matrix represents the full polarization response of an object at a particular wavelength using a Stokes vector basis. Given a set of four linearly independent input beams onto the object and the associated outputted optical responses of each beam, the 4×4 Mueller matrix relations follow as:

$$\mathbf{M}\begin{bmatrix} S_{i1} & S_{i2} & S_{i3} & S_{i4} \end{bmatrix} = \begin{bmatrix} S_{o1} & S_{o2} & S_{o3} & S_{o4} \end{bmatrix}$$

For our device, our illumination basis $\mathbf{P}$ is based on horizontal, vertical, diagonal and right-circular polarization states:

$$P_1 = \begin{bmatrix} 1 \\ 0 \\ 0 \\ 0 \end{bmatrix}, \quad P_2 = \begin{bmatrix} 0 \\ 1 \\ 0 \\ 0 \end{bmatrix}, \quad P_3 = \begin{bmatrix} 0 \\ 0 \\ 1 \\ 0 \end{bmatrix}, \quad P_4 = \begin{bmatrix} 0 \\ 0 \\ 0 \\ 1 \end{bmatrix}$$

Their description in terms of Stokes vectors is:

$$S_1 = \begin{bmatrix} 1 \\ 1 \\ 0 \\ 0 \end{bmatrix}, \quad S_2 = \begin{bmatrix} 1 \\ -1 \\ 0 \\ 0 \end{bmatrix}, \quad S_3 = \begin{bmatrix} 1 \\ 0 \\ 1 \\ 0 \end{bmatrix}, \quad S_4 = \begin{bmatrix} 1 \\ 0 \\ 0 \\ 1 \end{bmatrix}$$

Conversion between our illumination basis and the Stokes vectors follows a linear operation using the matrix $\mathbf{A}$:

$$S = \mathbf{A}P, \quad P = \mathbf{A}^{-1}S, \quad \mathbf{A} = \begin{bmatrix} 1 & 1 & 0 & 0 \\ 1 & -1 & 0 & 0 \\ -1 & -1 & 2 & 0 \\ -1 & -1 & 0 & 2 \end{bmatrix}$$

For analysis, we use polarization filters also with the $\mathbf{P}$ basis. The Mueller matrix M response of the sample using our illumination and analyzer polarization bases can therefore be expressed as:

$$\mathbf{MA}\begin{bmatrix} P_{i1} & P_{i2} & P_{i3} & P_{i4} \end{bmatrix} = \mathbf{M}\begin{bmatrix} S_{i1} & S_{i2} & S_{i3} & S_{i4} \end{bmatrix} = \begin{bmatrix} S_{o1} & S_{o2} & S_{o3} & S_{o4} \end{bmatrix}$$

$$= \mathbf{A}\begin{bmatrix} P_{o1} & P_{o2} & P_{o3} & P_{o4} \end{bmatrix} = \mathbf{AC}, \quad \mathbf{M} = \mathbf{ACA}^{-1}$$

Where $\mathbf{C}$ is the calibrated measurement matrix and $\mathbf{C}_{ij}$ denotes the measured output intensity of polarization j with respect to the input polarization i.

S5 Calibration algorithm

S5.1 Neural network architecture

The neural network layers are specially designed to meet the unique requirements of our physical system and noise sources. For typical neural networks, strided convolution layers and/or pooling layers are used for downsampling the feature map during the forward process. However, such layers break the translation symmetry of the inputted data form, as a 2x downsampling will result in the feature map shifting by only half the amount of shift in the input. Thus, we do not use any of such layers, and the network does not perform downsampling using large convolution kernels. Rather, a convolution kernel with size n and no padding decreases the feature map size by $n-1$. By stacking multiple such layers, the feature maps can be adjusted to suitable sizes.

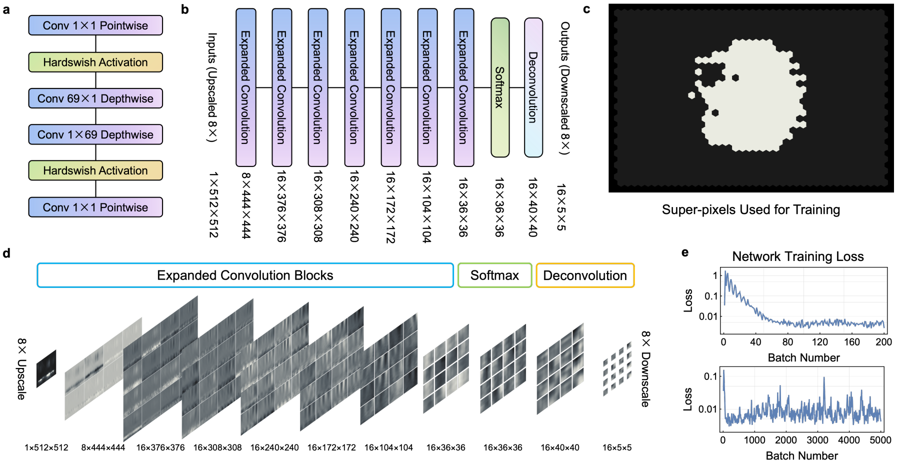

Fig. S11: Neural network architecture and training. a) Structure of an expanded convolution layer used within the network. This expanded layer contains four convolution layers and two hardswish activation layers. b) Architecture of the used neural network. The input is upsampled followed by downsampling, and one softmax and deconvolution layer are each specified after the expanded convolution layer blocks. c) Super-pixel positions used from the training dataset comprising images of an aluminum wafer, highlighted in white. d) Structure of the neural network visualized with an example input. e) Training loss curve of the neural network over 200 batches and 5000 batches.

To mitigate overfitting issues and speed up training, we design the expanded convolution block shown in Figure S11a. The convolution operation of the block follows a four-step pattern, in the order of a 1×1 pointwise convolution that increases the channel dimension by $4\times$, two depthwise convolutions with kernels of 1×69 and 69×1, and a final pointwise convolution.

This design significantly reduces the parameter count of the model, resulting in faster and more robust training. A stack of 7 expanded convolution blocks reduces the feature map size from the input size of 512 to 36 (Figure S11b), which corresponds to 4.5 pixels in the original image, the estimated upper bound for the point spread and translation caused by noise. Apart from the new convolution design, Hardswish activation functions [2] are used in the network to encourage sparse learning of features.

Upon processing with 7 expanded convolution blocks, the outputted data form comprises a set of channels each containing a feature map of some learnt features of the input image that will be used for generating the sampling kernels $\mathbf{K}$. Ideally, this correction vector would be a sum of various kernels that represent different point spread functions, such that it would be necessary to use a deconvolution (transposed convolution) layer to generate the correction vector (Figure S11b). However, this point spread function should not depend on the input intensity levels, which influences the average value of the final feature map. To deal with this constraint, a softmax layer is added prior to the deconvolution layer to normalize the feature maps (Figure S11b), which limits the sum of all the elements to 1 and removes the effect of overall brightness. For the deconvolution layer, we use a kernel size of 37 and padding of 16, which slightly increases the output size to 40. After this layer, the output results are downsampled with average pooling to 5, which produces $\mathbf{K}$ with the correct dimension.

S5.2 Neural network training and evaluation

We implemented the neural network architecture using Apache MXNet[3], built with CUDA 11.3 support. This enables dramatic speedup in training using graphic cards.

To obtain training data, we use an aluminum reflection mirror as our sample and place different combinations of spectral filters at the light source and polarization filters in the incidence or reflected beam path in the 4f system Fourier plane. These filters are in addition to the pinhole polarization filters at the illumination and analyzer planes. We used horizontal, vertical, 45°, 135° and right-circular polarizers with 5 band-pass filters. We consider a combination of wavelengths, polarization filter types, and polarization filter positions that yield 42 full wafer calibration images for training, details for each filter combination are shown in Table S5.

The training target is computed based on an ideal setup in which the Mueller matrix from a perfectly aligned reflective sample is:

$$M_0 = R \begin{pmatrix} 1 & -\cos 2\varphi & 0 & 0 \\ -\cos 2\varphi & 1 & 0 & 0 \\ 0 & 0 & \sin 2\varphi \cos \Delta & \sin 2\varphi \sin \Delta \\ 0 & 0 & -\sin 2\varphi \sin \Delta & \sin 2\varphi \cos \Delta \end{pmatrix}$$

	Band-pass Filter	Polarizer
1	550 nm-600 nm	linear polarizer 0°
2	600 nm-650 nm	linear polarizer 0°
3	650 nm-750 nm	linear polarizer 0°
4	700 nm-750 nm	linear polarizer 0°
5	750 nm-800 nm	linear polarizer 0°
6	None	linear polarizer 0°
7	550 nm-600 nm	linear polarizer 135°
8	600 nm-650 nm	linear polarizer 135°
9	650 nm-750 nm	linear polarizer 135°
10	700 nm-750 nm	linear polarizer 135°
11	750 nm-800 nm	linear polarizer 135°
12	None	linear polarizer 135°
13	550 nm-600 nm	linear polarizer 45°
14	600 nm-650 nm	linear polarizer 45°
15	650 nm-750 nm	linear polarizer 45°
16	700 nm-750 nm	linear polarizer 45°
17	750 nm-800 nm	linear polarizer 45°
18	None	linear polarizer 45°
19	550 nm-600 nm	linear polarizer 90°
20	600 nm-650 nm	linear polarizer 90°
21	650 nm-750 nm	linear polarizer 90°
22	700 nm-750 nm	linear polarizer 90°
23	750 nm-800 nm	linear polarizer 90°
24	None	linear polarizer 90°
25	550 nm-600 nm	None
26	600 nm-650 nm	None
27	650 nm-750 nm	None
28	700 nm-750 nm	None
29	750 nm-800 nm	None
30	None	None
31	550 nm-600 nm	quarter wave plate 0°
32	600 nm-650 nm	quarter wave plate 0°
33	650 nm-750 nm	quarter wave plate 0°
34	700 nm-750 nm	quarter wave plate 0°
35	750 nm-800 nm	quarter wave plate 0°
36	None	quarter wave plate 0°
37	550 nm-600 nm	quarter wave plate 45°
38	600 nm-650 nm	quarter wave plate 45°
39	650 nm-750 nm	quarter wave plate 45°
40	700 nm-750 nm	quarter wave plate 45°
41	750 nm-800 nm	quarter wave plate 45°
42	None	quarter wave plate 45°

Table S5: Polarization and band-pass filter combination used in calibration data.

R is the polarization-independent reflection coefficient and φ and Δ are functions of the incident beam angle onto the sample and the complex refractive index of aluminum [4]. We can compute the measurement matrix by combining M_0 with the Mueller matrices of the inserted polarizers. Due to illumination variability in the experimental system, only the 232 super-pixels shown in Figure S11c are used for fitting.

To make better use of the limited training data and to reduce over-fitting, we augment the data set by creating linear superpositions of the original 42 calibration images with random weights and weigh the training target images accordingly.

During training, we use the squared average of the difference between the outputted and targeted image pixel values as the loss function. We use a Nvidia RTX 3090 GPU for neural network training and an Intel core i7-8700K CPU for evaluation. The network is trained using the Adam optimizer[5] with a starting learning rate of 0.001 and a batch size of 16. With our hardware, it takes 50 minutes to train 1000 batches. The training loss curve is shown in Figure S11e. This curve show that the network training converges after 80 batches, and an extended training of 5000 batches shows no visible improvement. The average inference/calibration time for 1200 super-pixels using the neural network on a CPU takes 15 minutes. With our pre-generated calibration kernels, the calibration process takes only 200 ms on the same computer.

S6 Thin film measurement

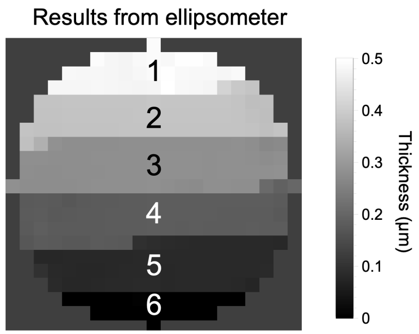

Fig. S12: Thin film silicon dioxide thickness map based on ellipsometer measurement.

For measurement of the reference film thickness, we used a Wollam RC2 Ellipsometer on the wafer with a grid size of 0.45 cm. The measured thickness map is shown in Figure S12.

To perform thin film analysis, the experimental measurement matrices are first calibrated with our neural network and then converted to Mueller matrices using the methods above. To use these calibrated measurements to fit silicon dioxide thickness at each super-pixel position, we minimize absolute error between the measured Mueller matrices and those computed using a thin film model. To model our air-silicon dioxide-silicon system, we use the transfer matrix method to compute the reflected polarization and spectral response as a function of input polarization. The simulated incidence beam is oriented at a

45 degree angle relative to the wafer. The index of refraction of air is specified to be 1.0 and the indices of refraction for the other two dispersive materials are shown below as a function of wavelength[6] (Table S6).

Wavelength/μm	n_{Si}	n_{SiO2}
0.552261	4.07697	1.45981
0.577786	4.00146	1.45878
0.603207	3.9403	1.45794
0.62852	3.88891	1.45713
0.65372	3.84534	1.45642
0.678805	3.8081	1.45583
0.70377	3.77884	1.45522
0.728611	3.7527	1.45464
0.753326	3.73023	1.45413
0.77791	3.71129	1.45375
0.802361	3.69031	1.45325

Table S6: Index of refraction used for fitting.

To demonstrate the need for proper calibration, we show a fitting of the thin film thicknesses without calibration. The results are shown in Figure S13 and display thicknesses that are highly erroneous compared to ground truth values. We also consider a calibration method based on the fitting of a global point spread function for all points in all super-pixels. The results for a 5×5 point spread function, fitted using the same training data, are also shown in Figure S13 and indicate that such basic calibration methods are also insufficient to reconstruct an accurate thickness map. To demonstrate the utility of our multi-generation kernel approach, we perform calibration using first generation kernels directly generated from the neural network (Figure S14). The results indicate that without the final optimization, some of the super-pixels are not functional and produces output with too much noise for fitting.

Figure S15 shows absolute error maps for all elements of the fully calibrated Mueller matrices for all wavelengths and super-pixels, as measured for the silicon dioxide-on-silicon wafer analyzed in the main text.

References

[1] Jean Paul Hugonin and Philippe Lalanne. Reticolo software for grating analysis, 2021.

[2] Andrew Howard, Mark Sandler, Bo Chen, Weijun Wang, Liang-Chieh Chen, Mingxing Tan, Grace Chu, Vijay Vasudevan, Yukun Zhu, Ruoming Pang, Hartwig Adam, and Quoc Le. Searching for mobilenetv3. In *2019 IEEE/CVF International Conference on Computer Vision (ICCV)*, pages 1314–1324, 2019.

[3] Tianqi Chen, Mu Li, Yutian Li, Min Lin, Naiyan Wang, Minjie Wang, Tianjun Xiao, Bing Xu, Chiyuan Zhang, and Zheng Zhang. Mxnet: A

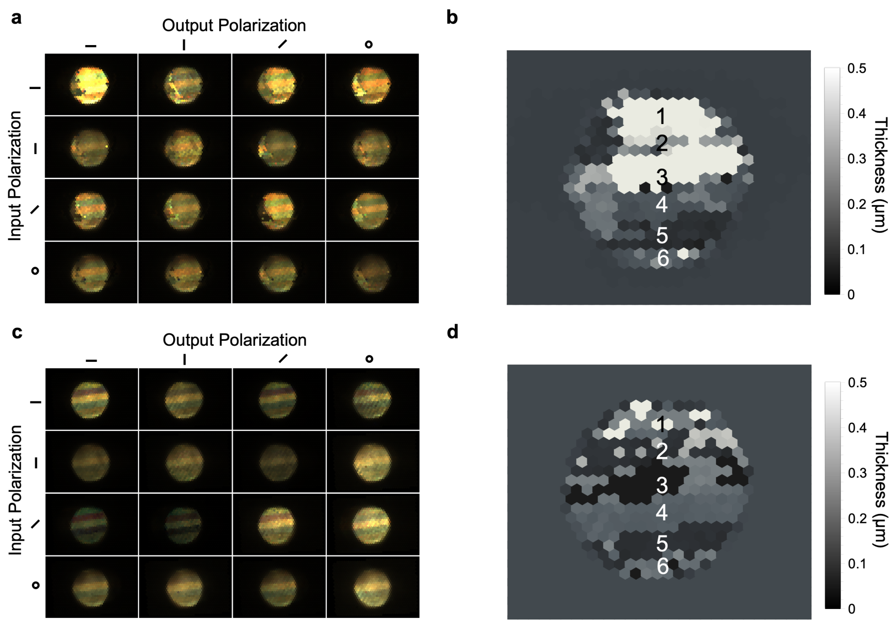

Fig. S13: Thin film fitting without calibration and using point spread function filtering technique. a) Directly obtained measurement matrix data from the 4" wafer comprising silicon dioxide thin films on a silicon wafer. b) Uncalibrated silicon dioxide thickness map based on the best-fit thin film model. c) Calibrated measurement matrix data from the silicon dioxide-silicon sample using a global fitted point spread function calibration method. d) Calibrated silicon dioxide thickness map based on the best-fit thin film model from (c).

flexible and efficient machine learning library for heterogeneous distributed systems. *ArXiv*, abs/1512.01274, 2015.

[4] Angus Macleod. Polarization in optical coatings. In Jose M. Sasian, R. John Koshel, and Richard C. Juergens, editors, *Novel Optical Systems Design and Optimization VIII*, volume 5875, page 587504. International Society for Optics and Photonics, SPIE, 2005.

[5] Diederik P. Kingma and Jimmy Ba. Adam: A method for stochastic optimization. *CoRR*, abs/1412.6980, 2015.

[6] Mikhail N. Polyanskiy. Refractive index database. https://refractiveindex.info.

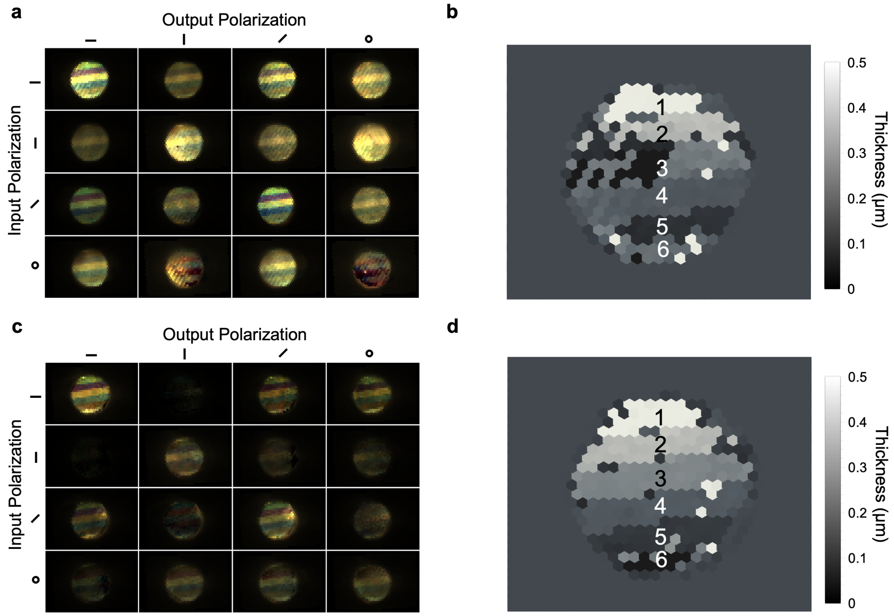

Fig. S14: Thin film fitting using first and second generation calibration kernels. a) Calibrated measurement matrix data from the 4" wafer comprising silicon dioxide thin films on a silicon wafer using first generation correction kernels. b) Corresponding calibrated silicon dioxide thickness map based on the best-fit thin film model. c) Calibrated measurement matrix data from the 4" wafer comprising silicon dioxide thin films on a silicon wafer using second generation correction kernels. d) Corresponding calibrated silicon dioxide thickness map based on the best-fit thin film model.

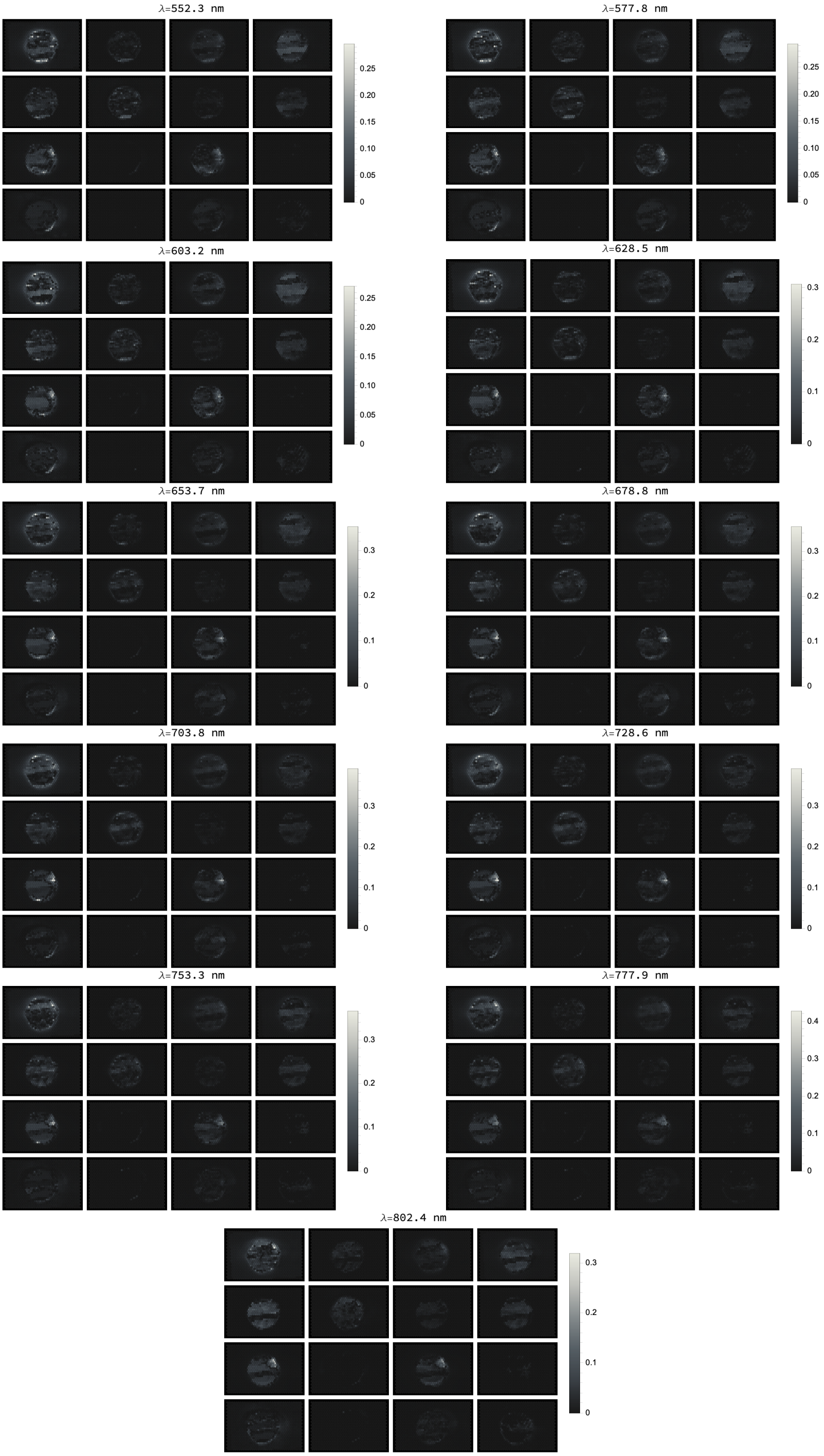

Fig. S15: Error map of Mueller matrices elements
